# Deep Learning Enables Superior Photoacoustic Imaging at Ultralow Laser Dosages

**DOI:** 10.1002/advs.202003097

**Published:** 2020-12-21

**Authors:** Huangxuan Zhao, Ziwen Ke, Fan Yang, Ke Li, Ningbo Chen, Liang Song, Chuansheng Zheng, Dong Liang, Chengbo Liu

**Affiliations:** ^1^ Research Laboratory for Biomedical Optics and Molecular Imaging CAS Key Laboratory of Health Informatics Shenzhen Institutes of Advanced Technology Chinese Academy of Sciences Shenzhen 518055 China; ^2^ Department of Radiology Union Hospital Tongji Medical College Huazhong University of Science and Technology Wuhan 430022 China; ^3^ Research Center for Medical AI CAS Key Laboratory of Health Informatics Shenzhen Institutes of Advanced Technology Chinese Academy of Sciences Shenzhen 518055 China; ^4^ Shenzhen College of Advanced Technology University of Chinese Academy of Sciences Shenzhen 518055 China

**Keywords:** deep learning, multitask residual dense networks, optical‐resolution photoacoustic microscopy, ultralow laser dosage

## Abstract

Optical‐resolution photoacoustic microscopy (OR‐PAM) is an excellent modality for in vivo biomedical imaging as it noninvasively provides high‐resolution morphologic and functional information without the need for exogenous contrast agents. However, the high excitation laser dosage, limited imaging speed, and imperfect image quality still hinder the use of OR‐PAM in clinical applications. The laser dosage, imaging speed, and image quality are mutually restrained by each other, and thus far, no methods have been proposed to resolve this challenge. Here, a deep learning method called the multitask residual dense network is proposed to overcome this challenge. This method utilizes an innovative strategy of integrating multisupervised learning, dual‐channel sample collection, and a reasonable weight distribution. The proposed deep learning method is combined with an application‐targeted modified OR‐PAM system. Superior images under ultralow laser dosage (32‐fold reduced dosage) are obtained for the first time in this study. Using this new technique, a high‐quality, high‐speed OR‐PAM system that meets clinical requirements is now conceivable.

## Graphical abstract

A multitask residual dense network (MT‐RDN) is proposed to meet the challenges in opticalresolution photoacoustic microscopy (OR‐PAM) translational research when laser dosage is confined. The MTRDN integrates multisupervised learning, dualchannel sample collection, and a reasonable weight distribution. Superior images under ultralow laser dosage (32‐fold reduced dosage) are obtained. Using this new technique, OR‐PAM that meets clinical requirements is conceivable.

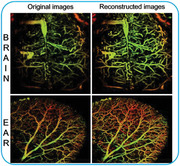

## 1 Introduction

Photoacoustic imaging is a rapidly growing biomedical imaging modality that images biological samples at multiple scales from organelles to organs.^[^ ^1^, ^2^ ^]^ This technology achieves anatomical, functional, and molecular imaging in situ and in real time with endogenous and exogenous contrasts. In recent years, many endogenous methods and exogenous contrast agents have been found and developed for photoacoustic imaging, which have greatly expanded the application range of this technology.^[^ ^3^, ^4^, ^5^, ^6^, ^7^, ^8^, ^9^ ^]^ Optical‐resolution photoacoustic microscopy (OR‐PAM) is a unique implementation of photoacoustic imaging where the spatial resolution is as fine as a micrometer or even sub‐micrometer.^[^ ^1^, ^10^, ^11^, ^12^ ^]^ OR‐PAM is specifically suitable for observing microvascular level biological processes and has been used in a wide range of preclinical studies, such as tumor angiogenesis, neurology, and ophthalmology.^[^ ^1^, ^13^, ^14^, ^15^, ^16^, ^17^, ^18^ ^]^

Over the past few years, the imaging speed of OR‐PAM has been improved by multiple folds.^[^ ^16^, ^19^, ^20^ ^]^ However, the maximum imaging speed achieved is still not adequate for many applications, such as brain‐wide neuronal activity study,^[^ ^21^ ^]^ where the imaging speed must be a minimum of ten to several hundred times faster than existing methods. Increasing the speed of photoacoustic imaging has been a long‐standing goal for researchers. An increase in the imaging speed requires a laser source that is capable of delivering laser pulses at high repetition rates. However, when the laser is working at a higher repetition rate, the per pulse energy of the laser pulse is reduced to maintain the laser dosage delivered to the biological tissue within the safety standard limits. The reduced pulse energy leads to low resultant photoacoustic signals, which leads to poor image quality. In addition, when multiple wavelengths are used for molecular or functional imaging, more restrictions are imposed on the per pulse laser energy, leading to a reduction of the signal‐to‐noise ratio (SNR) in the photoacoustic images. Downsampling techniques, where images are acquired sparsely over spatial distribution, are often used in photoacoustic imaging to reduce the number of data acquisitions (i.e., number of laser pulses) in exchange for a higher laser pulse energy to improve the SNR. However, the downsampling process also reduces the image quality due to the compromised spatial resolution. Thus, new methods to improve image quality while increasing the photoacoustic imaging speed and maintaining the safety of biological tissues are needed to expand the scope of photoacoustic imaging applications in both the preclinical and clinical worlds.

Due to hazard limitations placed on the laser dosage deposited in biological tissues, the only currently plausible way to improve the SNR while increasing the speed is through image and signal processing, i.e., so‐called information mining. Various image and signal processing algorithms have been developed in OR‐PAM to improve the image quality, such as image denoising,^[^ ^22^, ^23^ ^]^ compressed sensing,^[^ ^24^ ^]^ and vascular filtering.^[^ ^25^, ^26^ ^]^ While these algorithms have progressively improved the quality of OR‐PAM imaging successfully, the extent of the improvement using image‐based postprocessing methods has been limited. The image denoising algorithms^[^ ^23^ ^]^ may work well in simulations or phantoms, but improving the imaging quality of living samples is extremely difficult due to the complexity of biological tissues. Compressed sensing algorithms^[^ ^24^ ^]^ have been demonstrated to obtain superior reconstruction performance from undersampling data, but the reconstructed image is often suboptimal. Vascular filtering algorithms have been shown to improve the image quality based on mathematical operations, but inevitably resulted in vascular distortions.^[^ ^27^ ^]^ Most importantly, each algorithm proposed to improve one aspect of the image quality, but they all need to be combined to achieve overall high‐quality image reconstruction. However, when multiple algorithms are combined, the image reconstruction speed becomes slow because of the predominantly sequential operation, and the combined algorithm may not produce the desired improvement since each method makes its own underlying assumption about the data.

Deep learning (DL) is a class of machine learning techniques that uses multilayered artificial neural networks for the automated analysis of signals or data.^[^ ^28^, ^29^, ^30^, ^31^ ^]^ Convolutional neural networks (CNNs), composed of a convolution layer and a nonlinear operator, are a popular embodiment of DL technique. The CNN fits nonlinear equations by machine learning rather than manually providing equations for the image processing methods. The results using CNN have exceeded the performance of many traditional nonlinear image processing algorithms in photoacoustic imaging.^[^ ^32^, ^33^, ^34^ ^]^ These DL approaches are thus highly suitable to meet the challenges encountered in high‐speed photoacoustic imaging, i.e., achieving a high image quality and high SNR even at lower pulse laser energy. In this study, we propose a multitask residual dense network (MT‐RDN) to achieve superior quality imaging. The MT‐RDN has three subnetworks for multisupervised learning. Each subnetwork employs a residual dense network (RDN), and the weights for each subnetwork are allocated to achieve the best image reconstruction at each level. The MT‐RDN with three subnetworks achieves the required image denoising, super‐resolution, and vascular enhancement simultaneously for optimal photoacoustic imaging.

Currently, several DL methods exist for photoacoustic imaging,^[^ ^32^, ^33^, ^34^, ^35^ ^]^ where each method implements just one function, such as image denoising or super‐resolution. However, no technique has successfully achieved high‐quality imaging at low pulse laser energy (i.e., a low laser dosage) for the purpose of high‐speed imaging. Our multitask DL method addresses these issues and simultaneously realizes image denoising, super‐resolution, and vascular enhancement through multisupervised learning. The image denoising overcomes the low image SNR caused by the low per pulse laser energy. The image super‐resolution ensures the resolution of the image when downsampling is applied to reduce the overall laser dosage and increase the imaging speed. The vascular enhancement further improves the image quality without distorting the blood vessels. Furthermore, we obtained two photoacoustic imaging datasets with two different wavelengths, which were applied to the DL process. Each wavelength is sensitive to specific information in the imaging sample, and the information at each wavelength complements each other. The algorithm integrates the outcomes of the two datasets by a reasonable weight distribution to achieve further improved image quality. To the best of our knowledge, this improved result has never been achieved using other DL imaging methods. The MT‐RDN approach proposed in this study achieves superior image reconstruction results compared to the state‐of‐the‐art DL networks employed in photoacoustic imaging, such as U‐net and RDN.^[^ ^32^, ^34^ ^]^

In the next few subsections, we describe our DL technique for enhancing photoacoustic imaging. We also present a new imaging system that we have designed to take advantage of the proposed technique, which is very different from traditional OR‐PAM. We present our results and compare them with the existing traditional image processing method, i.e., the photoacoustic imaging vasculature enhancement filter (PAIVEF) method, and the state‐of‐the‐art DL methods, i.e., U‐net and RDN. Armed with the new technique, a high‐quality high‐speed OR‐PAM system that meets the needs in both preclinical and clinical imaging settings is now conceivable.

## 2 Experimental Section

### 2.1 Experimental System

**Figure 1:**
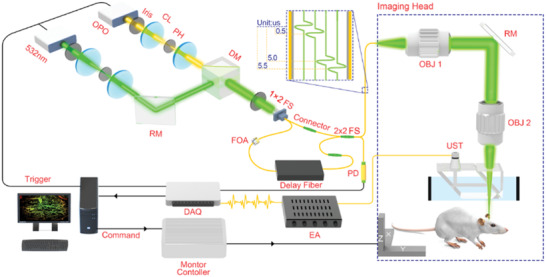
Schematic of the optical‐resolution photoacoustic microscopy (OR‐PAM) imaging system. CL: convex lens; FS: fiber beam splitter; FOA: fiber optic attenuator; RM: reflection mirror; OBJ: objective; EA: electronic amplifier; UST: ultrasonic transducer; PD: photodiode detector.

The schematic of the imaging system is shown in **Figure** **1**a. The 560 nm optical parametric oscillator (OPO) pulsed laser (NT‐242, Ekspla, Vilnius, Lithuania) and a 532 nm pulsed laser (GKNQL‐532, Beijing Guoke Laser Co., Beijing, China), both with a repetition rate of 1 kHz, were used as the illumination sources. The two output laser beams were each first reshaped by an iris (ID25SS, Thorlabs; 2 mm aperture size) and then focused by a condenser lens (LA1131, Thorlabs) before being spatially filtered by 50 µm pinholes (P50C, Thorlabs). After filtering, the beams were transformed into collimated beams using two condenser lenses, which were converged by a dichroic mirror (HIM025‐51‐45, Daheng Optics). The dichroic mirror reflected the 532 nm light and transmitted the 560 nm light. The converged laser beam was launched into a 1 × 2 multimode fiber beam splitter (Shenzhen IH Optics Co., Ltd., Shenzhen, China) with a core diameter of 50 µm. One output (Port 1) of the beam splitter's light was directly connected to one input of a 2 × 2 multimode fiber beam splitter (Shenzhen IH Optics Co., Ltd., Shenzhen, China) for high‐dosage photoacoustic imaging. The other output (Port 2) of the beam splitter's laser was attached to a fiber optic attenuator (FOA) to attenuate laser energy to achieve low dosage photoacoustic imaging and then was attached to an ≈150 m delay multimode fiber, which acted as a delay (≈750 ns) to separate the photoacoustic signals of both high and low dosages at the detection end. The delayed laser pulse was then connected to the other input of the same 2 × 2 multimode fiber beam splitter. In addition to the delay of the fiber, a 5 µs trigger delay was applied to the 532 nm laser compared to the OPO laser to separate the photoacoustic signals of the two wavelengths. The 5 µs delay was optimal to ensure that the photoacoustic signals of the two wavelengths could be separated while guaranteeing that the trigger delay would result in the least changes in the blood oxygen level. Hence, accurate blood oxygen saturation values could be measured. At the output end of the 2 × 2 multimode fiber beam splitter, there were four laser pulses spanning a time lapse of ∼5.5 µs (Figure 1b). The four laser pulses were a 560 nm high laser energy, a 560 nm low laser energy, a 532 nm high laser energy, and a 532 nm low laser energy. One output of the 2 × 2 multimode fiber beam splitter was connected to the imaging head of OR‐PAM for imaging purposes, and the other output end of the 2 × 2 multimode fiber beam splitter was connected to a photodiode detector for monitoring the pulse energy fluctuations. There were two causes for using multimode fibers for OR‐PAM illumination in this study: 1) poor quality of OPO laser spot; 2) four laser pulses (a 560 nm high laser energy, a 560 nm low laser energy, a 532 nm high laser energy, and a 532 nm low laser energy) must be coupled into the fiber for every experiment. The use of single‐mode fiber would cause low system stability and low optical coupling efficiency. Therefore, multimode fibers were employed in this study to ensure high system stability for obtaining training data. Multimode fibers would affect the spatial resolution to a certain extent, but in ref. ^[^ ^36^ ^]^ the authors had proven that OR‐PAM imaging can still be achieved: that is, optical focusing was much smaller than acoustic focusing. In this study, the spatial resolution obtained was 19.8 µm (shown in Figure S1b in the Supporting Information), which also proved it. The details of the supporting information of OR‐PAM and experimental operations can be found in Section S1 and Figure S1 (Supporting Information).

### 2.2 Establishment of Image Reconstruction

**Figure 2:**
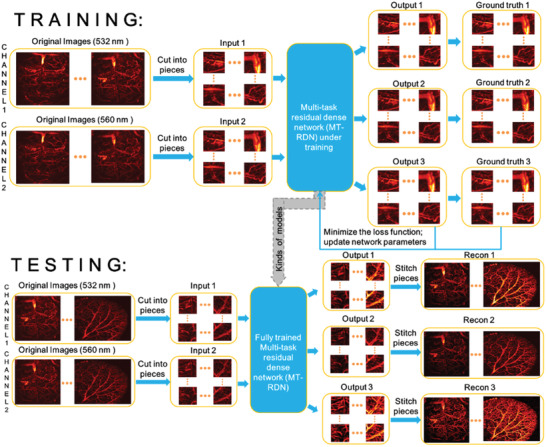
The overall framework of the proposed multitask residual dense network (MT‐RDN) method.

The overall framework of OR‐PAM image reconstruction based on the MT‐RDN method is shown in **Figure** **2**. This architecture was designed to solve the deteriorated image quality challenge caused by the low per pulse laser energy and undersampling during high‐speed imaging. During training, the original images (i.e., undersampling images obtained at low excitation laser energy) were collected at 532 and 560 nm wavelengths and were assigned to channel 1 and channel 2 in Figure 2, respectively. In this study, 2× and 4× undersampling images at the half per pulse laser energy of the ground truth (i.e., ANSI limit per pulse laser energy^[^ ^37^ ^]^) were used as the original images for the training. Next, the original images were cut into sectional pieces and were used as the input into the MT‐RDN network. The 2× undersampling images were cut into 100 × 100 pixels per piece, and the 4× undersampling images were cut into 50 × 50 pixels per piece. The selection of maximal 100 × 100 pixels in one sectional piece depended on the computing ability of the graphics processing unit of the computer used in this study. The sectional pieces in channel 1 and channel 2 were named Input 1 and Input 2, respectively, and were loaded into the MT‐RDN to obtain Output 1, Output 2, and Output 3. A detailed description of the MT‐RDN can be found in Section S2 (Supporting Information). Overall, the MT‐RDN had three subnetworks. The first subnetwork was used to process the data of Input 1 (i.e., 532 nm data) to obtain Output 1, and the second subnetwork was used to process the data of Input 2 (i.e., 560 nm data) to obtain Output 2. Outputs 1 and 2 were further combined and processed by subnetwork 3 to obtain Output 3. Hence, Output 3 contained complementary information of both 532 and 560 nm wavelengths. Finally, the differences between the outputs and ground truths were compared. The loss function was minimized, and the CNN‐related parameters were continuously updated to obtain the best training models. Corresponding to Outputs 1–3, Ground truths 1–3 were full sampling images obtained at the ANSI limit per pulse laser energy at 532 nm, full sampling images obtained at ANSI limit per pulse laser energy at 560 nm, and Ground truth 1 filtered by the PAIVEF method,^[^ ^26^ ^]^ respectively. Notably, Ground truth 3 was Ground truth 1 processed by the PAIVEF method to obtain enhanced image quality with a conventional non‐deep‐learning strategy. The PAIVEF method is an image processing algorithm based on Frangi's filter and is verified as an optimal vasculature enhancement filter which significantly improves image quality and causes less distortion in photoacoustic imaging.^[^ ^26^ ^]^ Similar to training, during testing, Input 1 and Input 2 were obtained by sectioning the original images (532 and 560 nm) into small subsections, which were then used as inputs into the MT‐RDN to obtain Output 1, Output 2, and Output 3. The subsections in each output were stitched together to obtain Recon 1, Recon 2, and Recon 3. The models were run on an Ubuntu 16.04 LTS (64‐bit) operating system equipped with a Xeon Silver 4110 central processing unit (CPU), 32GB memory and NVIDIA Quadro P5000 GPU (16GB memory).

To evaluate the advantages of the MT‐RDN over the existing state‐of‐the‐art supervised DL method, two state‐of‐the‐art neural network frameworks, namely, U‐net and RDN, were used for comparison. Among them, U‐net is the most commonly used CNN framework in general medical image reconstruction and segmentation,^[^ ^32^, ^38^, ^39^ ^]^ while RDN is verified to have a strong image reconstruction capability specifically in photoacoustic microscopy.^[^ ^34^ ^]^ For training these two networks, Input 1 and Ground truth 3 used in the MT‐RDN were used as the input and ground truth, respectively.

### 2.3 Animal Experiment

Ten healthy Balb/c mice (8 weeks, named the "Training Group") were selected to acquire 6696 training data (i.e., 6696 × 200 × 200 voxels of full sampling data were obtained), while one healthy Balb/c mouse (8 weeks, named the "Testing Group") was selected to acquire the testing data. For the ten mice in the Training Group, brain imaging was performed at wavelengths of 532 and 560 nm, while for the one mouse in the Testing Group, both brain and ear imaging experiments were performed at the two wavelengths. The acquired brain and ear imaging of the test mouse were used to assess the efficacy and the universal applicability of the proposed method.

The brain data in the Training Group included 1) 2× undersampling images using two wavelengths at 1/2 the ANSI limit per pulse laser energy; 2) 4× undersampling images using two wavelengths at 1/2 the ANSI limit per pulse laser energy; and 3) full sampling images using two wavelengths at the ANSI limit per pulse laser energy as ground truth.

The brain data in the Testing Group included 1) 2× undersampling images using two wavelengths at 1/2 the ANSI limit per pulse laser energy, named Data 1; 2) 4× undersampling images using two wavelengths at 1/2 the ANSI limit per pulse laser energy, named Data 2; and 3) full sampling images using two wavelengths at the ANSI limit per pulse laser energy, named Data 3, which were used as ground truth to compare with Recon data.

The ear data in the Testing Group included 1) 2× undersampling images using two wavelengths at 1/2 the ANSI limit per pulse laser energy, named Data 4; 2) 4× undersampling images using two wavelengths at 1/2 the ANSI limit per pulse laser energy, named Data 5; and 3) full sampling images using two wavelengths at the ANSI limit per pulse laser energy, named Data 6, which were used as ground truth to compare with the Recon data. Furthermore, to verify the effectiveness of the MT‐RDN with an even lower laser dosage, the following data on ear were obtained: 1) 2× undersampling images using two wavelengths at 1/3 the ANSI limit per pulse laser energy, named Data 7; 2) 2× undersampling images using two wavelengths at 1/4 the ANSI limit per pulse laser energy, named Data 8; and 3) full sampling images using two wavelengths at the ANSI limit per pulse laser energy, named Data 9, which were used as ground truth to compare with the Recon data.

### 2.4 Animal Handling

For brain imaging, a surgical procedure was performed to remove the scalp, while for ear imaging, no pretreatment was required. During imaging, the mice remained anesthetized using 1.5% isoflurane gas (Euthanex, Palmer, Pennsylvania) mixed with oxygen. Coupling gel was applied to the imaging area, and the imaging head of the OR‐PAM system was placed directly above it. All animal handling and experimental procedures conformed to a protocol approved by the Animal Study Committee of Shenzhen Institutes of Advanced Technology, Chinese Academy of Sciences.

### 2.5 Statistical Analysis

#### 2.5.1 Preprocessing of Data

All data were normalized to (0,1) before training, testing, and statistical analysis. Data used as Ground truth 3 were preprocessed by the PAIVEF method, as described in Subsection 2.2.

#### 2.5.2 Statistical Analysis of Quantitative Information

A statistical analysis was performed to evaluate the performance of MT‐RDN. Peak signal to noise ratio (PSNR) and structural similarity index (SSIM)^[^ ^40^ ^]^ were measured on the complete test data as follows

(1) $$\begin{array}{c} \mathrm{PSNR}=20\log_{10}\frac{\max\left(\mathrm{ref}\right)\sqrt{N}}{\left\|\mathrm{ref}\;-{\;\mathrm{rec}}_{2}^{2}\right\|} \end{array}$$

(2) $$\begin{array}{c} \mathrm{SSIM}=\frac{2\mu_{\mathrm{ref}}\mu_{\mathrm{rec}}+c_{1}}{\mu_{\mathrm{ref}}^{2}+\mu_{\mathrm{rec}}^{2}+c_{1}}\cdot \frac{2\sigma_{\mathrm{ref}\_\mathrm{rec}}+c_{2}}{\sigma_{\mathrm{ref}}^{2}+\sigma_{\mathrm{rec}}^{2}+c_{2}}\cdot \frac{\sigma_{\mathrm{ref}\_\mathrm{rec}}+c_{3}}{\sigma_{\mathrm{ref}}\sigma_{\mathrm{rec}}+c_{3}} \end{array}$$

where rec is the reconstructed image, ref denotes the reference image, and *N* is the total number of image pixels. The SSIM index is a multiplicative combination of the luminance term, the contrast term, and the structural term. *μ*_ref_,  *μ*_rec_ are the mean values of reconstructed images and reference images, respectively. *σ*_ref_,  *σ*_rec_ are the standard deviations (SDs) of reconstructed images and reference images, respectively. $\sigma_{\mathrm{ref}\_\mathrm{rec}}$ is the covariance of the reconstructed image and the reference image. *c*_1_,  *c*_2_,  *c*_3_ are non‐negative real numbers that specify the regularization constants for the luminance, contrast, and structural terms.

**Figure 3:**
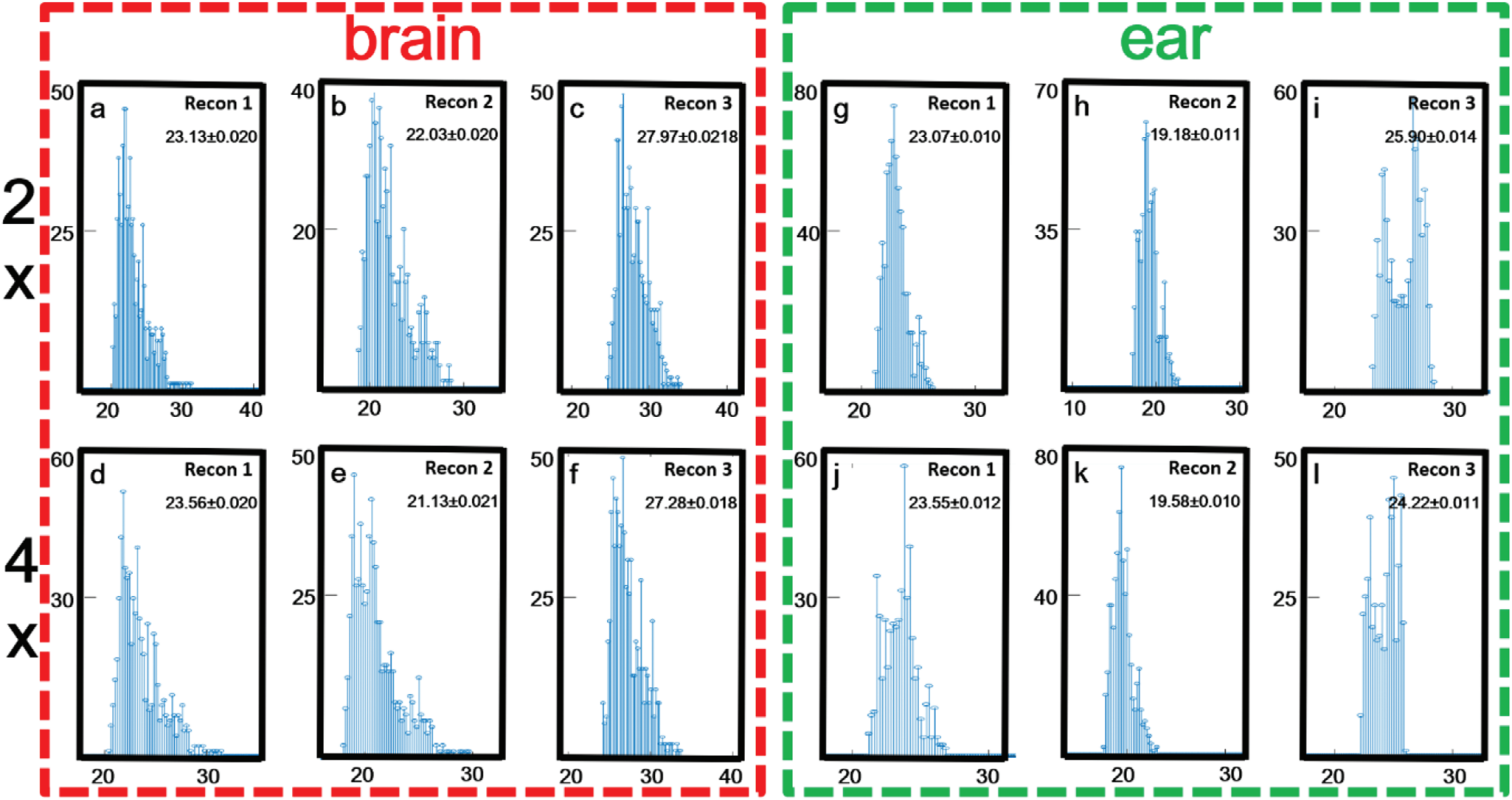
a–l) Quantitative analysis of peak signal to noise ratio (PSNR) of testing data: brain data are shown in the red dotted frame, and ear data are shown in the green dotted frame. The *x*‐coordinate represents the value of PSNR and the *y*‐coordinate represents the number of samples. Sample size = 720.

**Figure 4:**
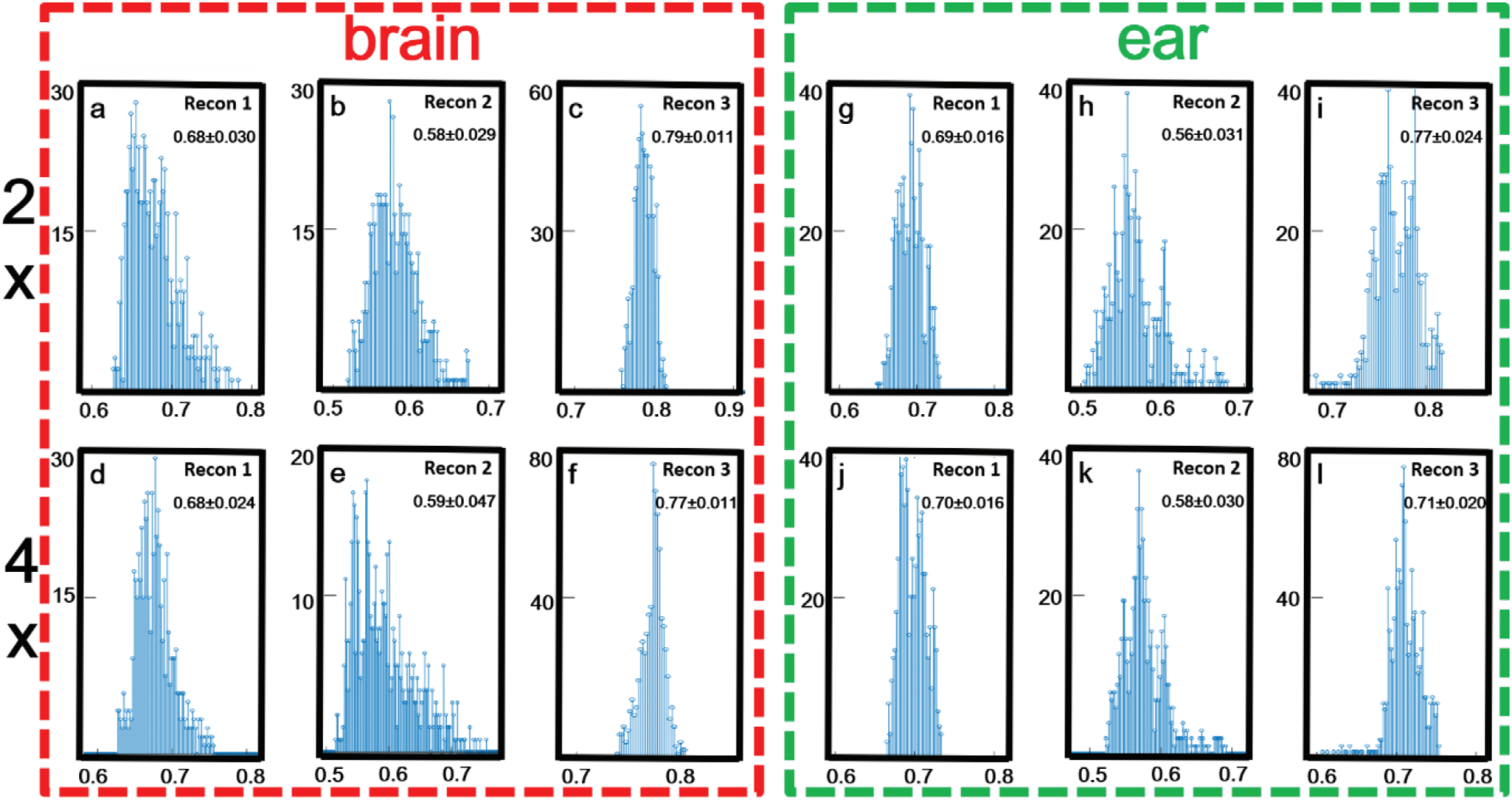
a–l) Quantitative analysis of structural similarity index (SSIM) of testing data: brain data are shown in the red dotted frame, and ear data are shown in the green dotted frame. The *x*‐coordinate represents the value of SSIM and the *y*‐coordinate represents the number of samples. Sample size = 720.

The histogram of PSNR and SSIM statistical distribution over the entire test dataset of brains (*n* = 720 pieces) and ears (*n* = 720 pieces) were presented to demonstrate the generalization performance of the proposed method. **Figures** **3** and **4** show the histogram of PSNR and SSIM statistical distribution on brain and ear test data, respectively. It could be seen that, even in the case of 4× downsampling data, the PSNR values of MT‐RDN method had reached a high level (the PSNR values of brain and ear were 27.28 ± 0.018 and 24.22 ± 0.011 (mean ± SD)). SSIM was mainly concentrated between 0.6 and 0.8. In 4× downsampling data, the SSIM values had reached a high level (the SSIM results of brain and ear were 0.77 ± 0.011 and 0.71 ± 0.020 (mean ± SD)). These indicators showed that the reconstruction results of MT‐RDN had a high degree of image reconstruction ability with respect to ground truth. The preprocessing and statistical analysis of data were implemented on MATLAB software (R2017a, Mathworks, Natick, MA).

## 3 Results and Discussion

**Figure 5:**
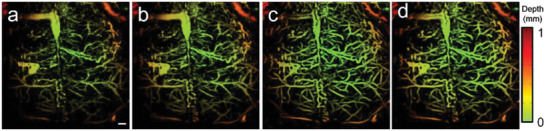
Comparison of the image quality before and after MT‐RDN. a) Input 1 of Data 2, b) Ground truth 1 of Data 2, c) Ground truth 3 of Data 2, d) Recon 3 of Data 2, scale bar = 0.5 mm.

The reconstruction results of Data 2 after using the MT‐RDN are shown in **Figure** **5**. Figure 5a–d shows the depth‐encoded images of Input 1, Ground truth 1, Ground truth 3, and Recon 3 of Data 2, respectively. Figures S3.1 and S3.2 (Supporting Information) show the complete results for comparison of all Input data, Recon data, and Ground truths for Data 1 and 2, respectively. The reconstructed blood vessels in Figure 5d have a high accuracy, as analyzed in Section S3 (Supporting Information). A distinct graininess can be seen in Figure 5a due to the low per pulse laser energy and downsampling. The reconstructed image in Figure 5d is significantly better than that in Figure 5a and is even better than the ground truth images (both Ground truth 1 and Ground truth 3), as shown in Figure 5b–d and Figure S3.2j,k (Supporting Information). The improved image quality of Recon 3 compared to Ground truth 1 and Ground truth 3 is presumably due to two reasons: 1) to obtain Recon 3, images without vascular distortion (i.e., Inputs 1 and 2) were used as inputs, and high SNR images were used as ground truth during the training process, which resulted in Recon 3 having a higher SNR than Ground truth 1 and significantly less vascular distortion compared to Ground truth 3; 2) the integration of complementary information between Input 2 (i.e., 560 nm data) and Input 1 during the MT‐RDN process added to the superiority of the algorithm. More descriptions and discussions can be found in Section S3 and Figures S3.1 and S3.2 (Supporting Information). Movie S1 (Supporting Information) provides an intuitional visualization of the image quality enhancement before and after the MT‐RDN process. Since the training data come from the map images, the map images during the entire training process of Data 2 are shown in Figure S3.3 (Supporting Information) to intuitively reflect the success of our training and testing. Furthermore, two key metrics, PSNR and SSIM, are used to further quantify and analyze the differences between Ground truth 3 and other images (Input 1, Input 2, Recon 1, Recon 2, Recon 3, and Ground truth 1). These two key metrics are shown in Tables S2 and S3 (Supporting Information).

**Figure 6:**
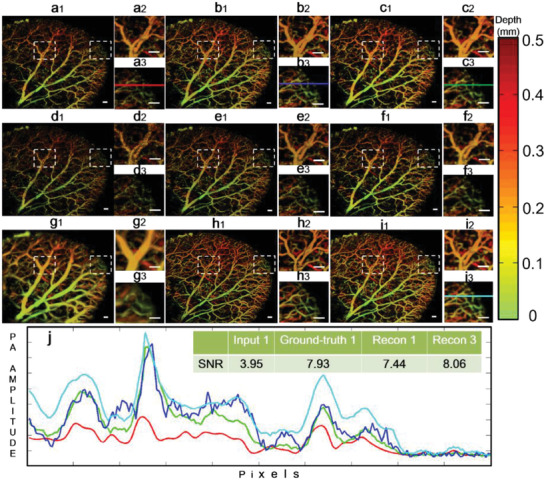
Validation of the universal applicability of the MT‐RDN by showing the key endpoints of the network when using Data 5 as the input. a–c) Input 1, Ground truth 1, and Recon 1, respectively; d–f) Input 2, Ground truth 2, and Recon 2, respectively; g–i) filtered image of Inputs 1 by PAIVEF, Ground truth 3, and Recon 3, respectively; and j) quantitative analysis of SNR of the selected area, where the selected areas are indicated by the solid lines in (a3), (b3), (c3), and (i3). Scale bar = 0.5 mm.

The training of the MT‐RDN was completed using the brain data in this study. Hence, the ear data, the vascular structure of which is quite different from the brain, were used to verify the universal applicability of the proposed method. **Figure** **6** shows the key end points of the MT‐RDN when using Data 5 as the input. Figure S3.4 (Supporting Information) shows the same results for Data 4. Figure 6a–i shows Input 1, Ground truth 1, Recon 1, Input 2, Ground truth 2, Recon 2, Input 1 filtered by PAIVEF, Ground truth 3 (reconstructed by another personal computer (PC) with a 128 GB CPU), and Recon 3, respectively. All the images are depth‐encoded to show the 3D information. The two areas indicated by the white dotted frames in the images are enlarged and shown in two separate figures. The image quality is significantly reduced in the undersampling images obtained by 1/2 the ANSI limit per pulse laser energy (Figure 6a,d). However, images reconstructed with the MT‐RDN demonstrate superior quality (Figure 6i). Compared to the PAIVEF method (Figure 6g), the MT‐RDN has the following advantages. 1) The PAIVEF algorithm causes image distortions when enhancing vascular signals. Consequently, the adjacent arteries and veins in the ear in Figure 6g cannot be separated. For the MT‐RDN, the arteries and veins can be easily distinguished, as shown in Figure 6i, and more importantly, significantly improved image quality, which is even better than Ground truth 1 and Ground truth 2, was obtained. 2) The PAIVEF method is computationally very expensive, and the memory requirement to reconstruct the full sampling image is very large. To reconstruct 4× the undersampling data (the size of the data is only 1/16 of the full sampling), PAIVEF took 94.5 s, and the memory requirement to reconstruct the full sampling image was beyond the maximum limit of the PC used in this study. The MT‐RDN has no such computation requirements and can perform image reconstruction in real time (≈0.45 s).

To quantitatively assess the advantages of the MT‐RDN, we plotted the signal intensity curves for the same region indicated by the color lines in Figure 6a3,b3,c3,i3, and the results are shown in Figure 6j. The different colors of the curves in Figure 6j correspond to the colors of the lines in Figure 6a3,b3,c3,i3. The upper right corner in Figure 6j indicates the SNR values of the color lines in Input 1, Ground truth 1, Recon 1, and Recon 3, which are 3.95, 7.93, 7.44, and 8.06, respectively. The SNR value of Recon 1 is significantly better than Input 1. However, the SNR value of Recon 3 is even higher, which is better than Ground truth 1. These results show the superior performance of the MT‐RDN, which improves the image quality even when the training samples are different from the testing samples. PSNR and SSIM are employed to quantify and analyze the difference between Ground truth 3 and other images (Input 1, Input 2, Recon 1, Recon 2, Recon 3, and Ground truth 1). **Tables** **1** and **2** show PSNR and SSIM values of each image, respectively. It can be determined from these tables that Recon 3 is superior in both two areas as compared to the other images, since both quantitative indicators of Recon 3 are better than others. The vascular morphology of the brain is quite different from that of the ear, thus establishing the universal applicability of the proposed MT‐RDN method. Movie S2 (Supporting Information) provides an intuitional visualization of the image quality enhancement before and after the MT‐RDN.

**Table 1:** Quantitative comparisons of PSNR between Ground truth 3 and other images of Data 5. The best‐performing value in each group is bolded

PSNR	Input 1	Input 2	Recon 1	Recon 2	Recon 3	Ground truth 1
Area 1	17.21	17.06	23.03	18.41	**25.15**	23.11
Area 2	18.54	18.70	24.01	20.12	**26.12**	25.09

**Table 2:** Quantitative comparisons of SSIM between Ground truth 3 and other images of Data 5. The best‐performing value in each group is bolded

SSIM	Input 1	Input 2	Recon 1	Recon 2	Recon 3	Ground truth 1
Area 1	0.5200	0.5038	0.7140	0.5690	**0.7556**	0.7061
Area 2	0.5318	0.5305	0.7061	0.5954	**0.7381**	0.7233

**Figure 7:**
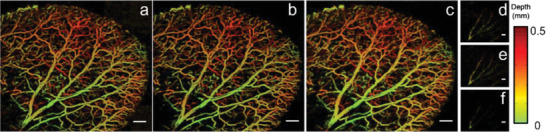
a) U‐net reconstruction results of the ear imaging data. b) RDN reconstruction results of the ear imaging data. c) Recon 3 of the MT‐RDN reconstruction results of the ear imaging data. d–f) The error maps of (a)–(c), respectively, with Ground truth 1. Scale bar = 1 mm.

To compare the quality of the reconstructed images between the state‐of‐the‐art DL techniques (U‐net and RDN) popular in photoacoustic imaging and our method, we provided an error map (i.e., residuals map), which is an intuitive and popular method used for evaluating the quality and distortion of the reconstructed images using CNN methods.^[^ ^41^, ^42^, ^43^ ^]^ When distortions increase, the patterns in the error maps become richer. The U‐net, RDN, and MT‐RDN reconstruction images of the ear data (Data 5) are shown in Figure 5a–c, respectively. Figure 5d–f shows the errors of full Figure 5a–c, respectively, when compared to Ground truth 1. The error map in **Figure** **7**f shows obvious advantages over Figure 7d,e. The error vascular signal in Figure 7f is significantly weaker than the error signal intensity in Figure 7d,e, which implies extremely small distortions in Figure 7c. Furthermore, the background noise signals in Figure 7f are at an ultralow level, indicating minimal fault extraction during reconstruction. Our method achieves high performance due to three main reasons: 1) it removes random noise using multiple channels; 2) it learns through a multisupervised learning strategy, thus avoiding the problem of gradient dispersion and making the data converge more easily and better; and 3) it has a reasonable weight distribution among the subnetworks, thus retaining more completed information when merging images of 532 and 560 nm wavelengths.

To explore the performance of the trained models at even lower per pulse laser energy, we imaged the mouse ear at 1/3 and 1/4 of the ANSI limit pulse energy. The results show the consistently accurate image reconstruction ability of the proposed method (see Section S4 and Figure S4 in the Supporting Information).

**Figure 8:**
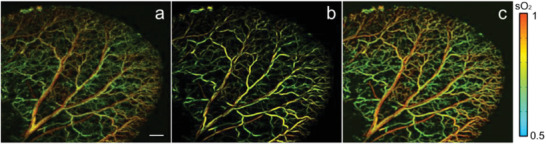
a–c) The sO2 maps use the original image, Frangi's filtered filter image, and Recon3 of MT‐RDN as the mask. Scale bar = 1 mm.

Vascular functional imaging is very important in disease management. For example, changes in the blood oxygen saturation of microvessels are key indicators of kidney disease. The poor quality visualization of the vascular structure caused by fast‐scanning OR‐PAM will inevitably affect the vascular functional imaging. Currently, two methods are used to improve imaging. 1) Reducing the sampling step size and averaging multiple data at the same time. Although this method greatly improves the image quality, it increases imaging time by multiple folds and is difficult to apply to real clinical cases. 2) Applying a filtered mask to sO2 images, as described in ref. ^[^ ^44^ ^]^. The filtered mask is obtained by processing the raw data of the blood vessel structures through a filtering algorithm. The commonly used filtering algorithms are a class of algorithms based on Frangi's filter. However, we believe that the images reconstructed by our proposed method are more suitable for deployment as masks. Figure 6a–c shows the sO2 maps of the original image, Frangi's filtered image, and the MT‐RDN reconstructed image (i.e., Recon 3 used as masks), respectively. Similar to the imaging results shown previously (Figure 6), our method in **Figure** **8**c shows an image without blood vessel distortion and no change in the sO2 value compared to the original image. A poor SNR and distinct graininess exist in Figure 8a, and although the vascular signals are smoothed in Figure 8b, several defects exist, including vascular distortion and changes in sO2 values compared to the original image. The results show that the MT‐RDN method can improve the image quality without affecting the calculation accuracy of sO2 values, which may provide a new method for the visualization of sO2.

The high‐quality images reconstructed by the proposed method address two key issues hindering the wide application of OR‐PAM, i.e., an excessive laser dosage and a low imaging speed. The DL method proposed in this paper overcomes both these challenges by achieving high‐quality image reconstruction even under the conditions of undersampling and ultralow per pulse laser energy, thus providing new insights into OR‐PAM imaging using low laser dosage. Furthermore, the experiments used brain data for training and ear data for testing, and did not make any assumptions about the correlation between the data while reconstructing them. Hence, the universal applicability of the algorithm was established, and the algorithm can be easily extended to other imaging samples. This is very important for certain applications such as brain and eye imaging. In these applications, the laser dosage permitted is further restrained due to the particularity of these organs compared to other organs such as skin. With our method, training can be performed first in other organs with higher laser dosages, while testing can be performed on the brain and eye with much lower laser dosages.

## 4 Conclusion

In photoacoustic imaging, the proposed MT‐RDN based on DL achieves high‐performance image reconstruction in terms of denoising, super‐resolution, and vascular enhancement. This network also has strong universal applicability, e.g., a model trained for the brain showed high performance for ear imaging. This method surpasses the most commonly used single‐supervised learning CNNs and hence has applications in not only photoacoustic imaging but also the general imaging field. Compared to ground truth, superior quality images with 16‐fold (1/4 ANSI limit per pulse energy, 2× undersampling) and 32‐fold (1/2 ANSI limit per pulse energy, 4× undersampling) reduced laser dosages were obtained when using MT‐RDN. The MT‐RDN is complemented in this study by a newly proposed OR‐PAM design, which scans samples quickly under ultralow laser dosages. These efforts bring photoacoustic imaging one step closer to clinical use. The trained models can be used to reconstruct high‐quality images in real time from undersampling images obtained at ultralow per pulse laser energy. This method effectively solves the image quality problem in high‐speed structure and functional OR‐PAM. Even though the MT‐RDN was trained on a custom‐made OR‐PAM system, the well‐trained MT‐RDN can be used on any OR‐PAM system. We believe the proposed method, which achieves high‐quality imaging within the ANSI power limit for OR‐PAM, will provide new insights in imaging diseased samples in the near future.

## Conflict of Interest

The authors declare no conflict of interest.

## Supporting information

Supporting Information

Supplement Movie 1

Supplement Movie 2
