# Supplementary material for: Deep Learning Enables Superior Photoacoustic Imaging at Ultralow Laser Dosages
Source: Adv Sci (Weinh). 2020 Dec 21;8(3):2003097. doi: 10.1002/advs.202003097 (PMC7856900; doi:10.1002/advs.202003097)
Supplement: Supplementary file 1 — Supporting Information [file ADVS-8-2003097-s001.pdf]

© 2020 Wiley-VCH GmbH

ADVANCED SCIENCE
Open Access

Supporting Information

for *Adv. Sci.,* DOI: 10.1002/advs.202003097

Deep Learning Enables Superior Photoacoustic Imaging at Ultra-low Laser Dosages

Huangxuan Zhao, Ziwen Ke, Fan Yang, Ke Li, Ningbo Chen, Liang Song, Chuansheng Zheng, Dong Liang*, and Chengbo Liu**

Supporting Information

Deep learning enables superior photoacoustic imaging at ultra-low laser dosages

Huangxuan Zhao, Ziwen Ke, Fan Yang, Ke Li, Ningbo Chen, Liang Song, Chuansheng Zheng, Dong Liang*, and Chengbo Liu**

1. The supplementary information of OR-PAM and experimental operations

As shown in Figure S1 (a), the output beam from the multi-mode fiber was collected into the imaging head, which was collimated by an inverted objective, and then focused by an identical objective to provide focused optical illumination into the tissue below the water dish. As shown in Figure S1 (b), the spatial resolution measured with a sharp metallic blade is ~19.8 μm. For *in vivo* imaging, the pixel step size is 10 μm. Both laser sources were operated at 1 KHz repetition rate. The full sampling data were obtained by laser energy reaching the biological tissue of ~800 nJ per pulse (likewise, 1/2, 1/3, and 1/4 the ANSI limit per pulse laser energy in this study corresponds to 400, 267, and 200 nJ per pulse, respectively), corresponding to an optical fluence of ~13.33 mJ/cm^2 on the skin surface (when focused ~200 μm below the skin surface), which conforms to the ANSI safety standards. Medical-grade ultrasound gel was evenly dispensed between the animal and water tank for acoustic coupling. Photoacoustic waves emitted from the tissue propagated through the water and the Combiner (mainly containing a rhomboidal prism and a triangular prism), totally reflected at the silicone oil layer filled between the rhomboidal and triangular prisms of the Combiner, and then detected by a 50-MHz ultrasonic transducer (V214-BC-RM, Olympus-NDT, Japan) placed on the top surface of the Combiner. The detected signals were pre-amplified using a commercial electrical amplifier (ZFL500LN-BNC, Mini-Circuits), digitized via a 200-MS/s data acquisition (DAQ) card (CS1422, GaGe), and then stored in a personal computer. A 6-mm diameter acoustic lens (45006, Edmund, acoustic NA: 0.43) was firmly attached to the bottom of the Combiner to enhance receiving sensitivity by confocally aligning the optical and acoustic foci. Volumetric OR-PAM images were obtained with mechanical raster scanning

via precisely programmed electrical scanners (PLS-85, Micos). The imaging speed of the OR-PAM system, limited by the speed of mechanical scanning, was ~0.5 frame (B-scan) per second.

2. Details of the MT-RDN convolutional neural network architecture

The proposed MT-RDN network is divided into three subnetworks, as shown in Figure S2. Each subnetwork employs an independent RDN framework [1] and is assigned a task of supervised learning. The three subnetworks are connected through weight distribution. First, Inputs 1 and 2 are used in Subnet 1 and 2 to reconstruct Recons 1 and 2, respectively. Recons 1 and 2 are summed according to the λ ratio and used as the input to Subnet 3 to reconstruct Recon 3. The red dashed boxes in the three subnetworks in Figure S2 are residual dense block (RDB) blocks, and the detailed operation of each RDB block is described in the expanded red dashed box on the right side of Figure S2. The forward process of this network is described below.

First, two under-sampled noisy images ($I_u^n, n = 1, 2$) are used as input (Inputs 1 and 2) into two subnetworks, Subnet 1 and 2, respectively, to extract the shallow features. Two convolutional layers are used to extract the shallow features:

$$\begin{cases} F_{-1}^n = \delta(W_{-1}^n * I_u^n + b_{-1}^n) \\ F_0^n = \delta(W_0^n * F_{-1}^n + b_0^n) \end{cases} \tag{S1}$$

where W_{-1}^n and W_0^n are weights and b_{-1}^n b_0^n are biases of the convolutional filters ($n = 1, 2$) of subnetworks 1 and 2, respectively. F_{-1}^n and F_0^n are the extracted shallow features, and δ is the nonlinear activation function.

Second, the shallow features are up-sampled through deconvolution layers. Since the input data I_u^n are under-sampled at R-fold acceleration, its size is only one part of the fully sampled data. The function of up-sampling is to restore it to the original size of the fully sampled data:

$$F_{up}^n = W_{up}^n *^{-1} F_0^n + b_{up}^n \tag{S2}$$

where W_{up}^{n} and b_{up}^{n} are the deconvolutional filters and biases, respectively; F_{up}^{n} is the up-sampled features, and $*^{-1}$ denotes the deconvolution operation.

Third, the up-sampled features go through D (D=4 in this study) RDBs for local feature fusion. The output F_d of the d-th RDB is expressed as follows:

$$F_{d}^{n} = f_{RDB,d}^{n}(F_{d-1}^{n}) = f_{RDB,d}^{n}(f_{RDB,d-1}^{n}(\ldots(f_{RDB,1}^{n}(F_{up}^{n}))\ldots)) \quad \text{(S3)}$$

where $f_{RDB,d}^{n}$ denotes the forward process of the d-th RDB belonging to subnetwork n. Each RDB comprises dense connections, local feature fusion, and local residual connections. Dense connections refer to the direct links that exist between each convolutional layer and the subsequent layers. The dense connections facilitate the transmission of local features across layers and make full use of features from preceding layers. All local features are concatenated and passed through a 1×1 convolutional layer to achieve local feature fusion. If each RDB has C (C=5 in this study) 3×3 convolutional layers, then the local feature fusion is defined as:

$$F_{d,LF}^{n} = W_{d}^{n}([F_{d-1}^{n}, F_{d,1}^{n}, \ldots, F_{c}^{n}, \ldots, F_{d,C}^{n}]) + b_{d}^{n} \quad \text{(S4)}$$

where W_{d}^{n} and b_{d}^{n} denote the weights and biases of the 1×1 convolutional layer in the d-th RDB and $[F_{d-1}^{n}, F_{d,1}^{n}, \ldots, F_{c}^{n}, \ldots, F_{d,C}^{n}]$ refers to the concatenation of the local features of the d-th RDB. The residual connections are introduced in the RDB to further improve the information propagation:

$$F_{d} = F_{up}^{n} + F_{d,LF}^{n} \quad \text{(S5)}$$

Fourth, these residual dense features from D RDBs are merged via global feature fusion (concatenation + 1×1 convolution):

$$F_{GF}^{n} = W_{GF}^{n}([F_{1}^{n}, F_{2}^{n}, \ldots, F_{D}^{n}]) + b_{GF}^{n}. \quad \text{(S6)}$$

Finally, we stack an up-scaling layer (in HR space) to obtain the finer resolution:

$$I_{HR}^{n} = \delta(W_{HR}^{n} * Upscale(F_{GF}^{n}) + b_{HR}^{n}). \quad \text{(S7)}$$

Here, we obtain the reconstruction of the two channels $I_{HR}^{n}, n = 1, 2$.

These two reconstruction results from the two channels (532 nm and 560 nm) are summed in λ proportions and are used as input to subnetwork 3:

$$I^{3} = \lambda I_{HR}^{1} + (1-\lambda) I_{HR}^{2} \quad \text{(S8)}$$

The forward process of subnetwork 3 is summarized as follows (n=3):

$$\begin{cases} F_{-1}^{3} = \delta(W_{-1}^{3} * I^{3} + b_{-1}^{3}) \\ F_{0}^{3} = \delta(W_{0}^{3} * F_{-1}^{3} + b_{0}^{3}) \\ F_{1}^{3} = f_{RDB,1}^{3}(F_{0}^{3}) \\ \cdots \\ F_{D}^{3} = f_{RDB,D}^{3}(F_{D-1}^{3}) \\ F_{GF}^{3} = W_{GF}^{3}([F_{1}^{3}, \cdots, F_{D}^{3}]) + b_{GF}^{3} \\ I_{HR}^{3} = \delta(W_{HR}^{3} * Upscale(F_{GF}^{3}) + b_{HR}^{3}) \end{cases} \quad \text{(S9)}$$

The forward process of subnetwork 3 is similar to subnetworks 1 and 2, with the exception that there is no up-sampling operation in this subnetwork.

The details of the convolutional layers are shown in Table S1[2, 3]. The mini-batch size was 2. The exponential decay learning rate [4] was used in all CNN-based experiments, and the initial learning rate was set to 0.0001 with a decay rate of 0.95. All the models were trained using the Adam optimizer [5] with parameters beta_1=0.9, beta_2=0.999, and epsilon=10^-8.

The loss function at the end is defined as follows:

$$J(\theta) = \alpha_{1} \frac{\sum_{i=1}^{n} (x^{(i)} - x^{(i)'})^{2}}{n} + \alpha_{2} \frac{\sum_{i=1}^{n} (y^{(i)} - y^{(i)'})^{2}}{n} + \alpha_{3} \frac{\sum_{i=1}^{n} (z^{(i)} - z^{(i)'})^{2}}{n} \quad \text{(S10)}$$

3. Results and Discussion of the brain and ear data in detail

Figures S3.1 and S3.2 show all images and quantitative analysis of Data 1 and Data 2 during image reconstruction. Figure S3.3 shows Maximum amplitude projection (MAP) images of Data 2, where each image in Figure S3.3 corresponds to Figure S3.2. In these figures, (a)-(c) are Input 1, Ground truth 1, and Recon 1; (d)-(f) are Input 2, Ground truth 2, and Recon 2; and (g)-(i) are filtered images of Input 1 by PAIVEF, Ground truth 3, and Recon 3, respectively.

All are depth-encoded to reflect the three-dimensional position information, and two areas (white dotted frames) are also enlarged for careful observation. From these figures, it can be determined that the image quality obtained by 1/2 the ANSI limit per pulse laser energy is significantly reduced in the under-sampling images (Figures S3.1 (a) and (d), Figures S3.2 (a) and (d)) compared to the images reconstructed by our method (Figures S3.1 (i) and S3.2(i)), where high image quality is still maintained.

To further quantify the advantages of the proposed method, the signal intensity curves, obtained from the same position, as shown in Figures S3.2 (a3), (b3), (c3), (h3) and (i3), are plotted in Figure S3.2 (j). The colours of the signal intensity curves in Figure S3.2 (j) correspond to the different colours in Figures S3.2 (a3), (b3), (c3), (h3) and (i3). The signal-noise ratio (SNR) is calculated in every image using the signal intensity curve. As shown in the upper right corner of Figure S3.2 (j), the SNR values in the selected region for Input 1, Ground truth 1, Recon 1, Ground truth 3, and Recon 3 are 3.64, 5.34, 6.44, 8.39 and 7.25, respectively. From these values, we can derive two important pieces of information: (1) our method improves the SNR by approximately 2× (Recon 3 vs. Input 1); and (2) the SNR of Recon 1 is higher than that of Ground truth 1. This result is expected, since a high laser energy per pulse will not only increase the blood vessel signal intensity, but also increase the noise signal intensity in deep tissues.

The vascular distortion due to image reconstruction is shown in Figure S3.2 (k) through a small selected region. Notably, for evaluating distortion, we deliberately selected blood vessels that are more likely to be distorted after reconstruction, thus verifying the reconstruction fidelity of the proposed method. The same region is selected across Figures S3.2 (b2), (h2) and (i2), and the signal strengths are plotted in Figure S3.2 (k). The different colours of the signal intensity curves in Figure S3.2 (k) represent the corresponding colours in Figures S3.2 (b2), (h2) and (i2), respectively. Corresponding to Figure S3.2 (k), severe vascular distortion can be observed in Figure S3.2 (h2): (1) two blood vessels shown in Figure

S3.2 (b2) are identified as three in Figure S3.2 (h2) after filtering; and (2) the blood vessels in Figure S3.2 (h2) are thinner after filtering. In comparison, no vascular distortion occurs in Figure S3.2 (i2) (i.e., two blood vessels in Recon 3), and the size of the blood vessels remains basically unchanged after reconstruction. Although the SNR value of the data reconstructed by our method is slightly lower than that of Ground truth 3, the accuracy of vascular enhancement is much higher, hence proving its advantage. It is worth noting two main reasons for the difference in quality between Input 1 and Input 2 in all images: 1. The excitation wavelengths used by Input 1 (532nm) and Input 2 (560nm) are different; 2. The quality of the excitation laser spot corresponding to Input 2 is inferior, which is due to the use of the OPO lasers. Therefore, if the problem of OPO laser spot quality was solved, the quality of the reconstruction images produced by the proposed method would be better.

All operations of Data 4 are the same as Data 5 (i.e., Ground truths 3 reconstructed by another personal computer (PC) with a 128 GB CPU), and the results are shown in Figures S3.4.

4. Analysis of the image reconstruction results of the imaging data under lower excitation laser dosage

The results of 2× under-sampled images obtained by 1/3 and 1/4 of the ANSI limit per pulse laser energy are shown in Figure S4 (a)-(g) and (h)-(n), respectively. The SNR is calculated for the same region in all of these images. The SNR values in Figs. S5 (a), (b), (c), (g) and (h), (i), (j), and (n) are 3.52, 7.09, 5.66, and 6.72 and 2.82, 7.22, 3.63, and 5.49, respectively. Thus, even at a very low SNR level, our method improves the SNR by approximately 2× and reconstructs high-quality images.

References

[1] Y. Zhang, Y. Tian, Y. Kong, B. Zhong, Y. Fu, "Residual dense network for image super-resolution", presented at *Proceedings of the IEEE Conference on Computer Vision and Pattern Recognition*, 2018.

[2] K. He, X. Zhang, S. Ren, J. Sun, "Delving deep into rectifiers: Surpassing human-level performance on imagenet classification", presented at *Proceedings of the IEEE international conference on computer vision*, 2015.

[3] X. Glorot, A. Bordes, Y. Bengio, "Deep sparse rectifier neural networks", presented at *Proceedings of the fourteenth international conference on artificial intelligence and statistics*, 2011.
[4] M. D. Zeiler, arXiv preprint arXiv:1212.5701 2012.
[5] D. P. Kingma, J. Ba, arXiv preprint arXiv:1412.6980 2014.

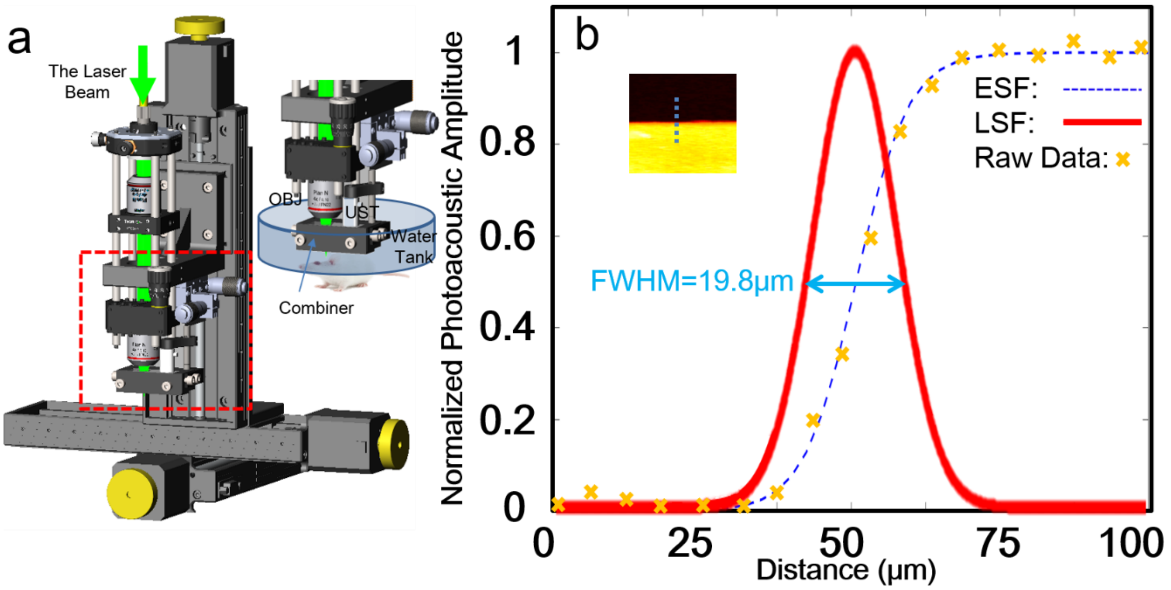

Figure S1. (a) Imaging head of the optical-resolution photoacoustic microscope (OR-PAM). OBJ: objective; UST: ultrasonic transducer; (b) Estimating the full width at half maximum (FWHM) of the original data using the edge of a sharp metallic blade. Green cross: original photoacoustic signal; blue dashed line: edge-spread function (ESF); red dash line: the first-order derivative of the ESF, representing the line spread function (LSF) along the scanning direction.

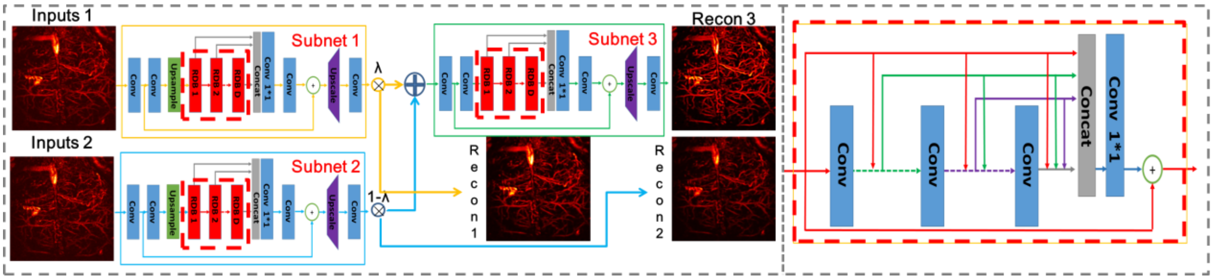

Figure S2. The flowchart of the MT-RDN. Conv: convolutional layer; RDB: residual dense block.

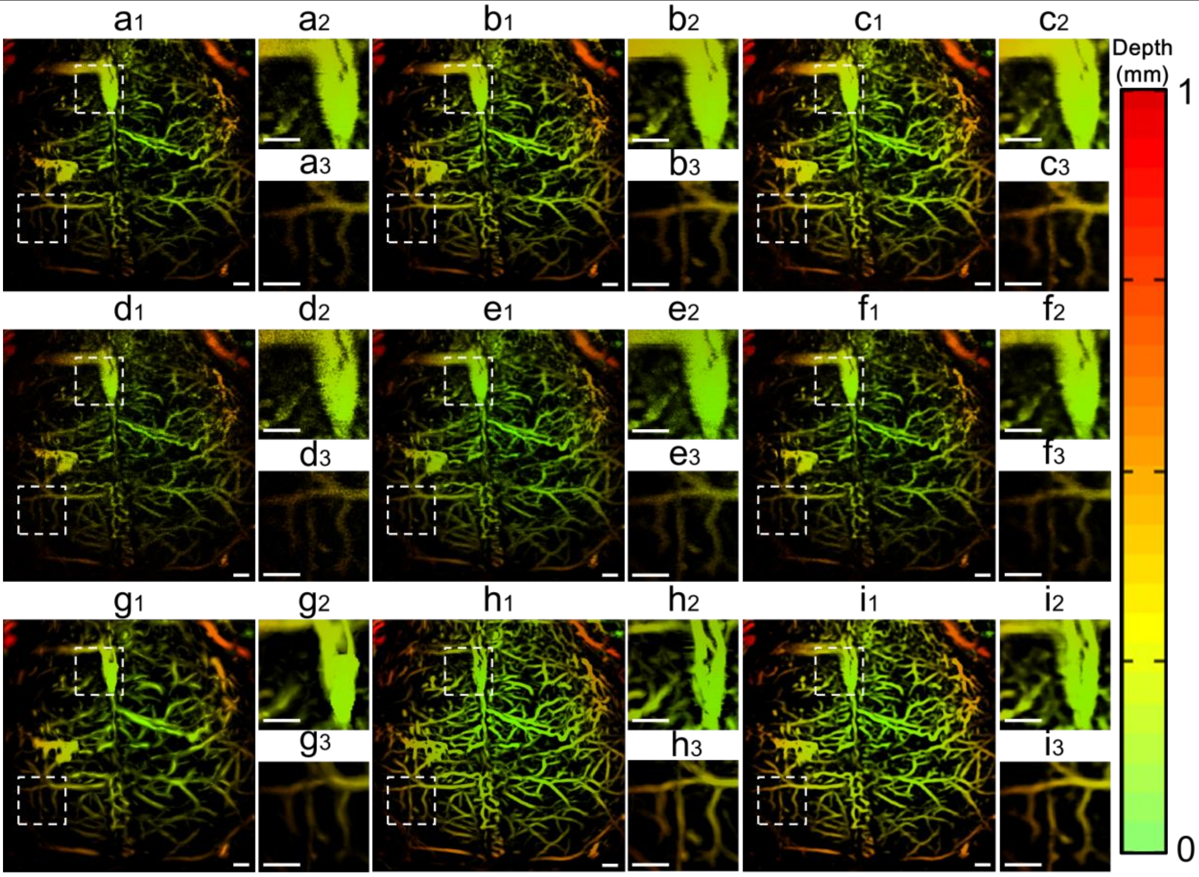

Figure S3.1. (a)-(c) Input 1, Ground truth 1, and Recon 1; (d)-(f) Input 2, Ground truth 2, and Recon 2; and (g)-(i) filtered image of Inputs 1 by PAIVEF, Ground truth 3, and Recon 3, respectively. Scale bar = 0.5 mm.

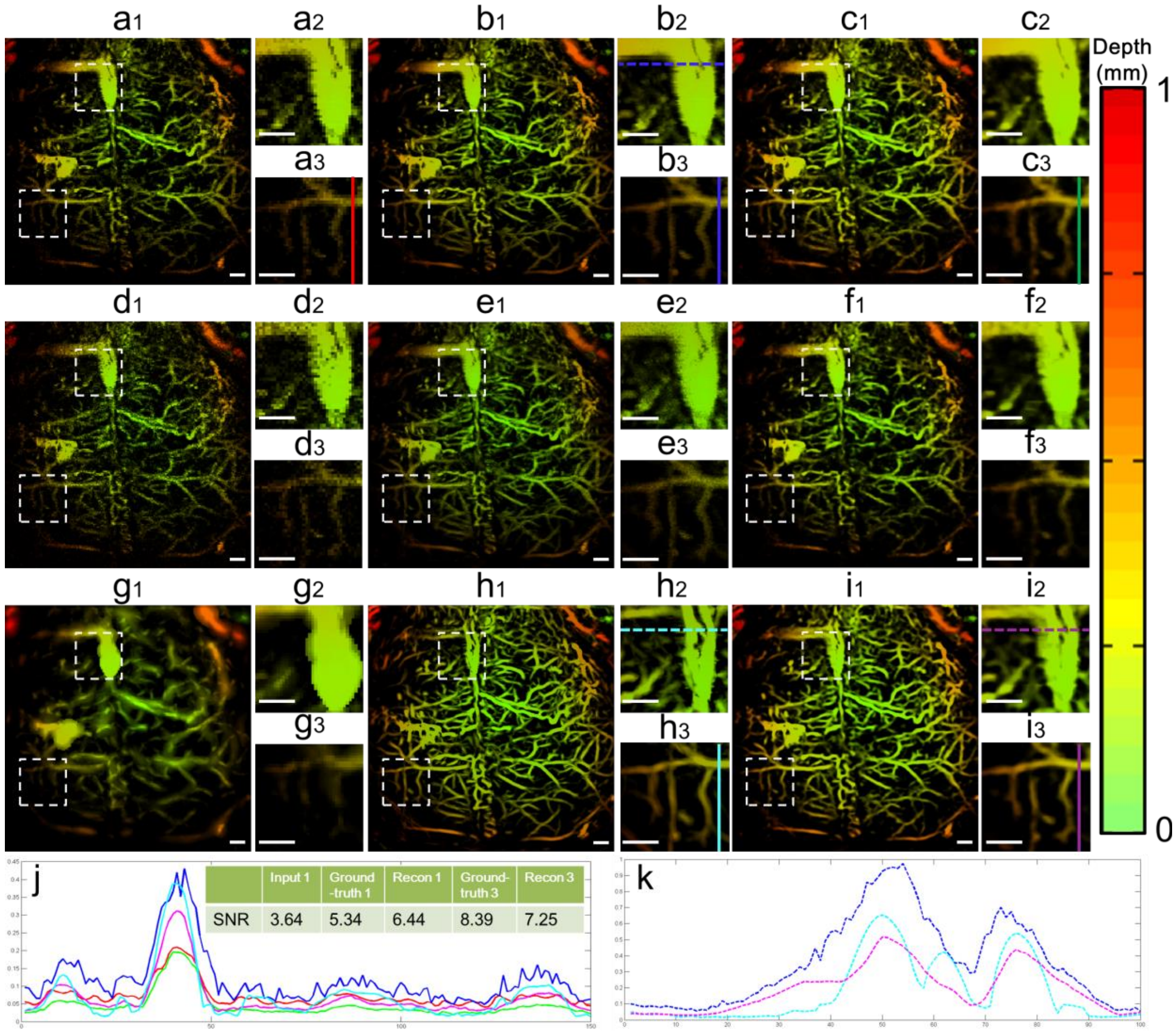

Figure S3.2. (a)-(c) Input 1, Ground truth 1, and Recon 1; (d)-(f) Input 2, Ground truth 2, and Recon 2; and (g)-(i) filtered image of Input 1 by PAIVEF, Ground truth 3, and Recon 3, respectively. (j) Quantitative analysis of the SNR of the selected area. The selected areas are indicated by solid lines in (a3), (b3), (c3), (h3) and (i3). (k) Quantitative analysis of the vascular distortion of the selected area. The selected areas are indicated by the dotted lines in (b2), (h2) and (i2). Scale bar = 0.5 mm.

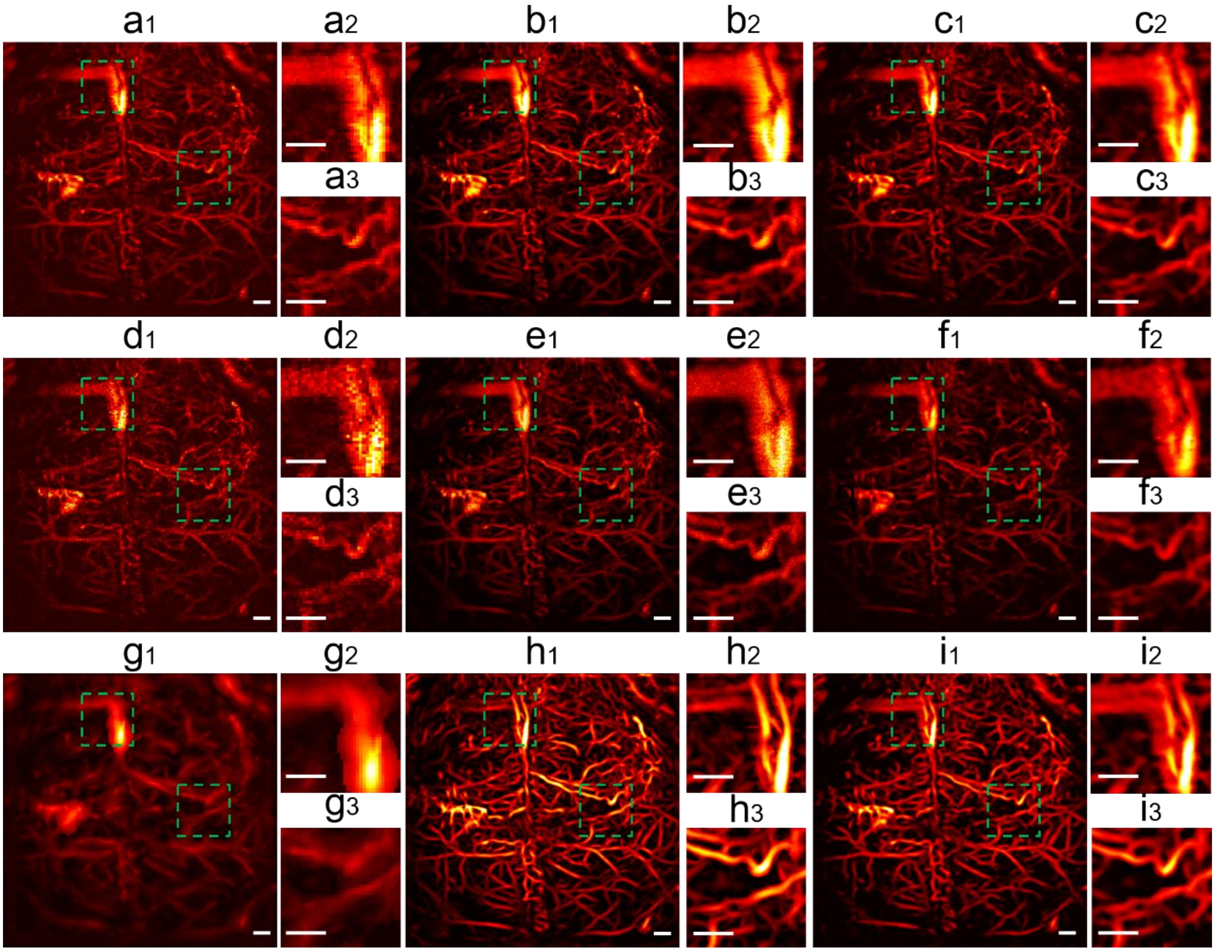

Figure S3.3. Maximum amplitude projection (MAP) images of DATA 2. (a)-(c) Input 1, Ground truth 1, and Recon 1; (d)-(f) Input 2, Ground truth 2, and Recon 2; and (g)-(i) filtered image of Input 1 by PAIVEF, Ground truth 3, and Recon 3, respectively.

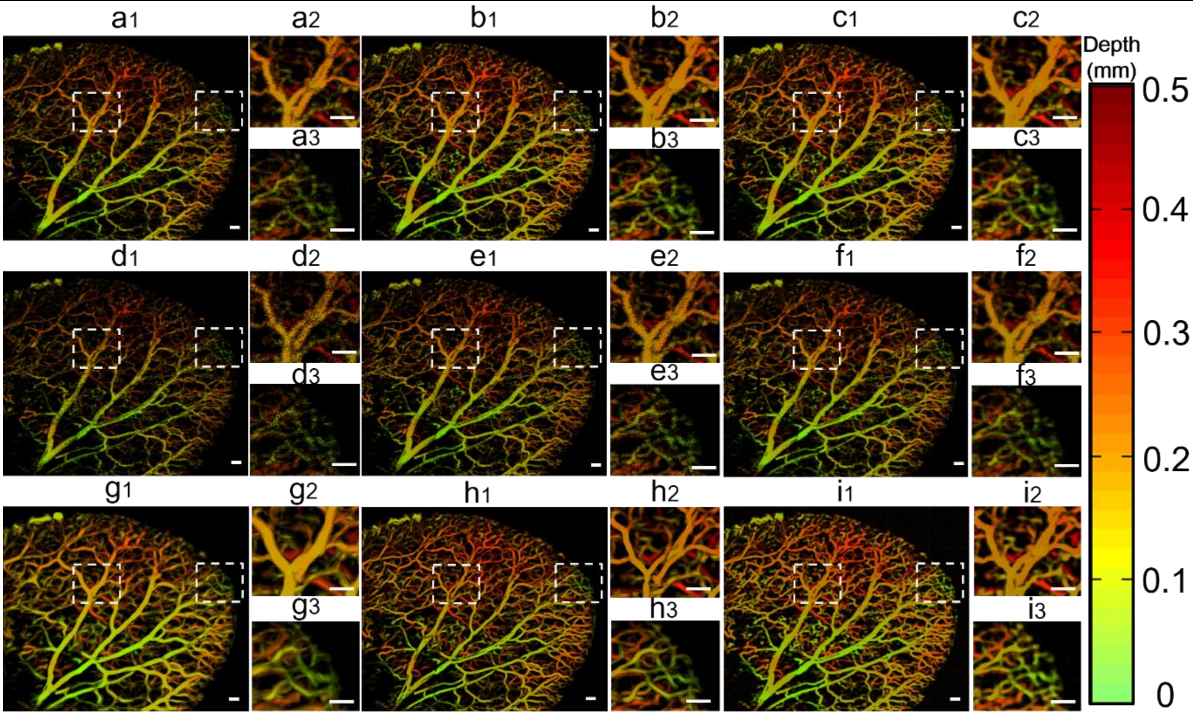

Figure S3.4. (a)-(c) Input 1, Ground truth 1, and Recon 1; (d)-(f) Input 2, Ground truth 2, and Recon 2; and (g)-(i) filtered image of Input 1 by PAIVEF, Ground truth 3, and Recon 3, respectively. Scale bar = 0.5 mm.

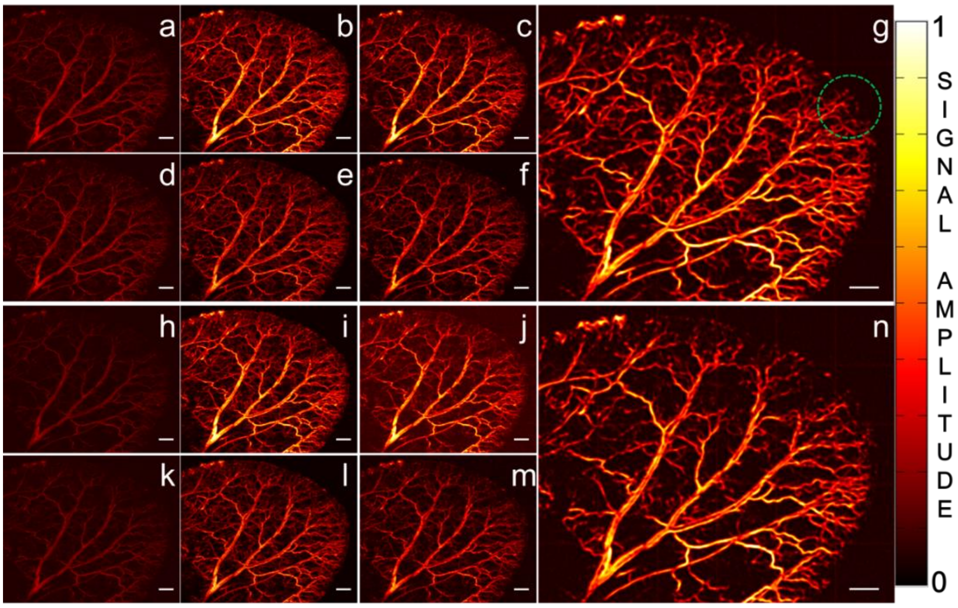

Figure S4. (a)-(g) are Input 1, Ground truth 1, Recon 1, Input 2, Ground truth 2, Recon 2, and Recon 3 of two under-sampling images obtained by 1/3 of the ANSI limit per pulse laser energy, respectively. (h)-(n) are Input 1, Ground truth 1, Recon 1, Input 2, Ground truth 2, Recon 2, and Recon 3 of two under-sampling images obtained by 1/4 of the ANSI limit per

pulse laser energy, respectively. Scale bar = 1 mm.

**Name**	**Kernel number**	**Kernel size**	**Weight initialization**	**Activation function**
**“Conv”**	32	3*3	He initialization[2]	RELU[3]
**“Conv 1*1”**	32	1*1	He initialization	None

TABLE S1. Details of the convolutional layers in the RDN.

PSNR	Input 1	Input 2	Recon 1	Recon 2	Recon 3	Ground truth 1
Area 1	21.97	20.83	22.02	19.25	**25.61**	19.76
Area 2	23.12	22.20	23.11	20.53	**26.90**	22.71

TABLE S2. Quantitative comparisons of PSNR between Ground truth 3 and other images of DATA 2. The best-performing value in each group is bolded.

SSIM	Input 1	Input 2	Recon 1	Recon 2	Recon 3	Ground truth 1
Area 1	0.6464	0.5902	0.6786	0.5538	**0.7911**	0.6094
Area 2	0.6685	0.6262	0.7046	0.5883	**0.8126**	0.6505

TABLE S3. Quantitative comparisons of SSIM between Ground truth 3 and other images of DATA 2. The best-performing value in each group is bolded.
